# Evaluation of Bile Salt Hydrolases, Cholesterol-Lowering Capabilities, and Probiotic Potential of *Enterococcus faecium* Isolated From Rhizosphere

**DOI:** 10.3389/fmicb.2019.01567

**Published:** 2019-07-16

**Authors:** Neelja Singhal, Anay Kumar Maurya, Shilpa Mohanty, Manish Kumar, Jugsharan Singh Virdi

**Affiliations:** ^1^Department of Microbiology, University of Delhi, New Delhi, India; ^2^Department of Biophysics, University of Delhi, New Delhi, India

**Keywords:** bile salt hydrolase, *Enterococcus faecium*, cholesterol-lowering, probiotics, hypo-cholesterolemia

## Abstract

Bile salt hydrolase (BSH) activity, hypo-cholesterolemic effect, and probiotic properties have been reported for *Enterococcus* strains isolated from animal and human gut and fermented foods but not for strains isolated from environmental niches, like aquatic and terrestrial plants, soil, and water. The present study is the first report on isolation of *Enterococcus faecium* from rhizospheric soils that harbor the *bsh* gene, remove cholesterol *in vitro*, and possess essential and desirable probiotic attributes. Fifteen samples were collected from different sites located in northern, southern, and central regions of India, of which five yielded pure colonies that were named LR2, LR3, ER5, LR13, and VB1. These were identified by 16S rRNA gene sequencing as *E. faecium* and evaluated for BSH activity, cholesterol-lowering potential *in vitro*, and probiotic properties. Our results indicated that all the strains were capable of surviving the harsh conditions of the gastrointestinal tract and did not harbor any of the virulence genes. Though all strains showed the presence of *bsh* and potential for cholesterol removal, *E. faecium* strain LR13 showed a remarkable cholesterol removal capability and vancomycin susceptibility and possessed most of the desirable and essential attributes of a probiotic. Hence, it seems to be a fairly promising probiotic candidate that needs to be further evaluated in *in vivo* studies, especially for its hypo-cholesterolemic potential.

## Introduction

Elevated serum cholesterol has become a major lifestyle-related disorder that results in high morbidity and mortality worldwide. Besides chemotherapeutics, natural measures like dietary interventions and, more recently, regular consumption of probiotics have been found to significantly improve the serum lipoprotein profiles. According to the World Health Organization (WHO), probiotics are viable microorganisms that, when consumed in sufficient amounts, deliver several health benefits on the consumer. Probiotics might exert hypo-cholesterolemic effect by several mechanisms: (i) by incorporating cholesterol in their cell membranes; (ii) by binding cholesterol on their cell surfaces, which inhibits formation of cholesterol micelles inside the intestine; and/or (iii) by assimilating cholesterol during growth, which reduces the amount of cholesterol available for intestinal absorption (Lye et al., 2010).

De-conjugation of the bile salts by bacterial bile salt hydrolase (BSH) has been reported as an important mechanism that is physiologically associated with the hypo-cholesterolemic effect of probiotics (Begley et al., 2006; Ha et al., 2006; Park et al., 2007). The WHO has recommended that along with capability for colonization of gastrointestinal (GI) epithelia and resistance to harsh GI conditions, BSH activity should be considered as one of the main criteria for selection of probiotics (FAO/WHO, 2002). Probiotic bacteria include several genera of lactic acid bacteria (LAB) like *Lactobacillus, Streptococcus, Lactococcus, Weissella, Pediococcus, Enterococcus* (Ehrmann et al., 2002), etc. *Enterococcus* is an important member of LAB, several strains of which have shown numerous health benefits like regulating the gut microbiota, alleviating obesity, allergy, irritable bowel syndrome and diarrhea (Franz et al., 2011), immunomodulation (Nissen et al., 2009), hypo-cholesterolemic effect (Ejtahed et al., 2011), etc. Over the last few years, some strains of *Enterococcus*, particularly vancomycin-resistant enterococci (VRE), were recognized as leading nosocomial pathogens harboring multiple genes for antimicrobial resistance and virulence factors (Ogier and Serror, 2008; Rosenthal et al., 2008; Franz et al., 2011; Arias and Murray, 2012). Thus, the genus *Enterococcus* has been conferred the dual status of a genus associated with both beneficial and pathogenic organisms. Recent studies have demonstrated that food-grade enterococci are safe and can be differentiated from the nosocomial pathogenic enterococcal strains (Montealegre et al., 2015). Currently, enterococcal strains that have been designated by regulatory agencies as commercial probiotics include *Enterococcus faecalis* Symbioflor^®^1 (SymbioPharm, Herborn, Germany), *Enterococcus faecium* SF68^®^ (NCIMB 10415, Cerbios-Pharma SA, Barbengo, Switzerland), and others (Serio et al., 2010; Franz et al., 2011).

Though studies have reported the BSH activity, hypo-cholesterolemic effect, and probiotic properties of *Enterococcus* strains isolated from animal and human gut (Hlivak et al., 2005; Hyrslova et al., 2016; Zhang et al., 2017) and fermented food products (Guo et al., 2016; Albano et al., 2018; Nami et al., 2019), the BSH activity and cholesterol-lowering ability of *Enterococcus* isolated from environmental niches, like aquatic and terrestrial plants, soil, and water, have not been reported. Bacteria isolated from environments in which bile salts are absent usually do not demonstrate BSH activity (Elkins et al., 2001; Moser and Savage, 2001; Ahn et al., 2003). Rhizosphere is a narrow region of the soil that is enriched with root secretions and soil microbes. *Enterococcus* strains isolated from rhizospheric samples might have some useful characteristics as they live in direct contact with soil and plant secretions and, co-evolve with plant pathogenic bacteria and fungi. Though a previous study reported several useful characteristics of rhizospheric-derived *E. faecium* strains (Klibi et al., 2012), to date, the cholesterol-lowering capability and probiotic potential of *E. faecium* isolated from rhizospheric soil have not been reported. To the best of our knowledge, our study is the first report on isolation and probiotic characterization of *E. faecium* isolates from rhizospheric soils, which also exhibited a significant cholesterol-lowering capability, *in vitro*.

## Materials and Methods

### Sample Processing and Isolation of LAB

A total of 15 rhizospheric soil samples were collected from different geographical regions of India (Hyderabad, Uttar Pradesh, Haridwar, and New Delhi) following the standard microbiological methods. Briefly, samples were collected in sterilized zipped plastic bags and transported to the laboratory and stored at 10°C. The samples were processed within a week of transport. One gram of the sample was added to screw-capped plastic bottles containing 99 ml of De Man, Rogosa and Sharpe (MRS) broth and incubated at 30°C for 48 h. One milliliter of culture from each bottle was re-inoculated into fresh MRS and again incubated for 48 h. This procedure was repeated thrice and the contents of each bottle were serially diluted and 100 μl of the diluted sample was spread on MRS agar plates that were incubated at 30°C till the colonies became visible. The isolated colonies were picked up randomly. Five cultures that yielded pure colonies were presumptively identified as LAB on the basis of growth on selective medium, Gram-positive staining, and catalase negative test (Holt et al., 1994; Day et al., 2001). The purified cultures were preserved at -80°C and sub-cultured at 30°C prior to use.

### Extraction of Genomic DNA and Identification of Isolates by 16S rDNA Sequencing

The five Gram-positive, catalase-negative LAB isolated from rhizospheric soil were designated as LR2, LR3, ER5, LR13, and VB1. For molecular identification of the LAB, genomic DNA was isolated using a commercial mdi Genomic DNA Miniprep Kit (Ambala Cantt, India). PCR amplification of the gene encoding 16S rRNA was carried out using the universal eubacterial primers in a Thermal Cycler (My Cycler^TM^, Bio-Rad, United States). Sequences of the forward and reverse primers were 5′AGAGTTTGATCCTGGCTCAG3′ and 5′ACGGCTACCTTGTTACGACTT3′, respectively. The PCR reaction mixture contained 1× PCR buffer (1.5 MgCl_2_, 1.5 mM KCl, 10 mM Tris–HCL, and 0.1% Triton X-100), 200 μM each of the four dNTPs, 12 U of Taq DNA polymerase (New England Biolabs, Ipswich, MA, United States), 10 pmol of forward and reverse primers, and 100 ng of genomic DNA in a final volume of 25 μl. The PCR reaction cycles were as follows: initial denaturation for 5 min at 95°C followed by 30 consecutive cycles of 30 s at 95°C, 30 s at 58°C, and 90 s at 72°C followed by a final extension for 10 min at 72°C. The PCR amplicons were electrophoresed on 1% agarose gels and visualized under UV light. The PCR amplicons were purified using QIA Quick Gel Extraction Kit (Qiagen, Hilden, Germany) and sequenced at a commercial facility (M/s Invitrogen India, Gurgaon, India). The gene sequences were searched for homology using BLASTN^1^.

### Temperature and Sodium Chloride Tolerance

The effect of different temperatures on growth and survival of *E. faecium* isolates was studied by incubating the isolates in MRS broth at 28, 37, and 45°C. Survival in salt was studied by growing the strains in MRS broth containing different concentrations of NaCl (0.5, 2, 3, 4, 5, 7, and 10%) at 37°C. In each case, the absorbance of the control and inoculated samples was measured at 600 nm after 24 h. The experiments were repeated for each isolate in triplicate and the mean values were reported ± SD.

### Assessment of Probiotic Characteristics

#### Resistance to Lysozyme

The resistance of *E. faecium* isolates to lysozyme was determined following a published method (Zago et al., 2011) and viable cell counts were determined at 0, 0.5, 1, and 2 h. The experiment was repeated for each isolate in triplicate and the mean values were reported ± SD.

#### Acid and Bile Tolerance

Acid tolerance was assessed by incubating the overnight grown *E. faecium* isolates in three separate flasks of MRS broth with pH adjusted to 1.5, 2, and 3 at 37°C. A 100-μl aliquot was drawn from each flask after 0, 1, and 3 h. Appropriate serial dilutions were made and incubated at 37°C for 18–24 h on MRS plates, and the number of colonies was calculated as log_10_ CFU/ml. For determining bile tolerance, overnight grown *E. faecium* strains were incubated in MRS broth containing different concentrations of bile salts (cholic and deoxycholic acid sodium salt; Sigma-Aldrich): 0.06, 0.125, 0.25, 0.5, and 1% (w/v). The cultures were incubated at 37°C, and absorbance was recorded at 600 nm after 24 h. Both the experiments were repeated for each isolate in triplicate, and the mean values were reported ± SD.

#### Resistance to Simulated Gastric Juice (Pepsin)

Simulated gastric juice was prepared by dissolving 0.3% (w/v) pepsin in saline (0.5%, v/v). The pH was adjusted to 3.0. Overnight grown LAB cultures were centrifuged, and the washed bacterial pellet was suspended in 4 ml of sterile NaCl (0.8%) solution. One milliliter of the cell suspension was mixed with 9 ml of gastric juice (pH 3) and vortexed for 15 s. The cultures were incubated at 200 rpm, and viable cell counts were determined at 0, 2, and 4 h at 37°C. The experiment was repeated for each isolate in triplicate, and the mean values were reported ± SD.

#### Cell Surface Hydrophobicity

The ability of the LAB to adhere to surfaces was assessed by measuring their cell surface hydrophobicity following the protocol described earlier (Lee et al., 2011). The experiment was repeated for each isolate in triplicate and the mean values were reported ± SD.

#### Auto-Aggregation and Co-aggregation

The auto-aggregation capability of *E. faecium* isolates was assessed using a published method (Juárez Tomás et al., 2005). The capability of *E. faecium* isolates for co-aggregation was quantified by measuring their co-aggregation with *Escherichia coli* NG9 following a published protocol (Golowczyc et al., 2007). The co-aggregation was expressed as the percentage reduction in the absorbance of the mixed suspension compared to the individual suspensions. Both the experiments were repeated for each isolate in triplicate, and the mean values were reported ± SD.

### Screening for Bile Salt Hydrolase Activity and the *bsh* Gene

For assessment of BSH activity, a sterile cork borer was used to punch wells of 6-mm diameter in 0.5% bile salt (cholic and deoxycholic acid sodium salt; Sigma-Aldrich, United States) supplemented MRS plates. Two hundred microliters of MRS broth containing overnight grown culture of *E. faecium* isolates was added to each well. Plates were kept at 37°C for 24–48 h and visually checked for appearance of white zone of precipitation around the wells.

Primers were designed to amplify the complete coding sequence (CCDS) of BSH by retrieving the whole genome sequence of *E. faecium* and the corresponding sequence of *bsh* from NCBI, along with some upstream and downstream regions of the gene. Sequences of the forward and reverse primers were BF 5′TGGAATGAGGAAAACTTTGGAG3′ and BR 5′CTGTTTTCTGGATCAACGA3′. The PCR reaction mixture and reaction conditions were the same as for amplification of gene encoding 16S rRNA, except the annealing temperature, which was kept at 55°C. The PCR amplicons were purified, sequenced, and analyzed for protein homology using the same methods as described for sequencing of the 16S rRNA gene. The CCDS of *bsh* of all the *E. faecium* isolates were translated using ExPASy^2^ and aligned using Clustal ‘Ω^3^.

#### Cholesterol Assimilation

The capability of *E. faecium* isolates to assimilate cholesterol in MRS broth supplemented with and without bile salts was assessed using a modified method (Rudel and Morris, 1973). Cholesterol was added to MRS broth supplemented with 6 mM sodium tauroglycocholate at a final concentration of 70 μg/ml. *E. faecium* strains were incubated in this medium at 37°C for 20 h. The samples were centrifuged; the pellet was washed with distilled water and kept at 80°C in an oven to obtain constant dry weight. One milliliter of the supernatant was added to 2 ml of 95% ethanol followed by 1 ml KOH (33%). The mixture was vortexed and heated at 37°C for 15 min. Then, 3 ml of *n*-hexane and 2 ml of distilled water were added and vortexed. The phases were allowed to separate at room temperature. Hexane layer was transferred to another test tube and evaporated at 90°C to obtain the residue. The residue was dissolved in 2 ml of *o*-phthalaldehyde (5 mg/ml) and mixed followed by the addition of 0.5 ml of concentrated sulfuric acid. The mixture was allowed to stand at room temperature for 10–15 min, and the absorbance of inoculated and un-inoculated samples was recorded at 570 nm. A standard curve of absorbance versus different concentrations of cholesterol in MRS (0, 3.91, 7.81, 15.63, 31.25, 62.5, 125, 250, and 500 μg/ml) was obtained. The rate of cholesterol assimilation by various isolates was calculated using the following formula:

$$\begin{array}{c} \mathrm{Cholesterol}\;\mathrm{assimilated}\;(\mu g/\mathrm{ml})=\mathrm{cholesterol}\;(\mu g/\mathrm{ml})\;\mathrm{at}\;0\;h-\mathrm{cholesterol}\;(\mu g/\mathrm{ml})\;\mathrm{after}\;20\;h \end{array}$$

The percent cholesterol assimilation was calculated using the formula:

$$\begin{array}{c} \%\;\mathrm{cholesterol}\;\mathrm{assimilated}=[\mathrm{cholesterol}\;\mathrm{assimilated}\;(\mu g/\mathrm{ml})/\mathrm{cholesterol}\;(\mu g/\mathrm{ml})\;\mathrm{at}\;0\;h]\times 100 \end{array}$$

The experiments were repeated for each isolate in triplicate and the mean percentage of cholesterol assimilation was reported ± standard deviation (SD).

### Biofilm-Forming Potential

The biofilm-forming potential of the enterococcal isolates was determined by crystal violet assay following the published protocols with slight modifications, at different pH values, *viz.*, 4, 5, and 6, and at different time points, *viz*., 24, 48, and 72 h (Stepanović et al., 2000; Sharma et al., 2018). Briefly, 50 μl of overnight grown *E. faecium* strains (∼1 × 10^9^ cells/ml) were inoculated in 1 ml of MRS broth, contained in 1.5 ml of polypropylene microcentrifuge tubes (Tarsons, United States). The bacteria were incubated at 37°C for different time periods, to allow the formation of biofilms. After the incubation period, medium was decanted and the microcentrifuge tubes were dried at 55°C for 30 min. One milliliter of 0.1% crystal violet (prepared in isopropanol:methanol:phosphate-buffered saline in the ratio of 1:1:18, v/v) was added to the microcentrifuge tubes and incubated at room temperature for 30 min. After this, crystal violet was removed, followed by two washings with 1 ml of sterile distilled water. Microcentrifuge tubes were further dried at 55°C for 30 min. The dye bound to the biofilm was dissolved in a 200-μl mixture of ethanol and acetone (4:1 v/v), and 100 μl of this mixture was added to a 96-well microtiter plate. Optical density was measured at 540 nm using an ELISA plate reader (Thermo Scientific, United States). Un-inoculated MRS broth was considered as the negative control. The cutoff OD (ODc) was defined as three standard deviations above the mean OD of the negative control. The strains were classified as strong, moderate, or weak biofilm producers based on the ODs of bacterial biofilms, using the following criteria:

$$\begin{array}{c} \mathrm{OD}>4\times \mathrm{ODc}\;\;\;\;\;:strong biofilm producer\\ \begin{array}{c} \;\;2\times \mathrm{ODc}<\mathrm{OD}\leq 4\times \mathrm{OD}:moderate biofilm producer\\ \mathrm{OD}\leq \mathrm{ODc}\geq 2\times \mathrm{ODc}\;:weak biofilm producer \end{array} \end{array}$$

The experiment was performed for each strain in triplicate and the average result ± SD was reported. The statistical significance was calculated using the Mann–Whitney *U* test with R statistical package. A *p*-value < 0.05 was considered as significant.

### Safety Evaluation

#### Hemolytic Activity and DNase Activity

The hemolytic activities of *E. faecium* isolates were determined using the published protocol (Pieniz et al., 2014). Production of the enzyme DNase was determined using the method suggested by Gupta and Malik (2007).

#### Antibiotic Susceptibilities

The antibiotic susceptibilities of *E. faecium* isolates were assessed using the Kirby Bauer disk diffusion method on Muller–Hinton (MH) agar plates. The antibiotic disks (HiMedia, India) for antibiotics that are commonly used for treatment of enterococcal infections were placed on the surface of agar, and plates were incubated at 37°C for 24 h. Results were interpreted as per the guidelines of the Clinical Laboratory Standards Institute (Clinical and Laboratory Standards Institute [CLSI], 2010).

#### Screening for Presence of Virulence Genes

Presence of virulence factors in the genomes of *E. faecium* isolates was investigated by PCR—amplification of the genes encoding eight major virulence factors, *viz.*, aggregation protein (*agg*), enterococcal surface protein involved in immune evasion (*esp*), extracellular metallo-endopeptidase (*gelE*), cytolysin (*cyl*), cell wall adhesins (*efaAfm*), chemotactic for human leukocytes and facilitate conjugation (*cpd, cob*, and *ccf*). The primer sequences and the PCR conditions were the same as described in previous studies (Eaton and Gasson, 2001; Vankerckhoven et al., 2004; Sabia et al., 2008) and are presented in Table 1.

**Table 1:** Primers used for detection of genes for virulence factors in *E. faecium* strains isolated from rhizosphere.

Virulence gene	Primer sequence	Amplicon size	References
*agg*	F:AAGAAAAAGAAGTAGACCAAC R:AAACGGCAAGACAAGTAAATA	153	Eaton and Gasson, 2001
*ccf*	F:GGGAATTGAGTAGTGAAGAAG R: AGCCGCTAAAATCGGTAAA	543	Eaton and Gasson, 2001
*cob*	F: AACATTCAGCAAACAAAGC R:TTGTCATAAAGAGTGGTCAT	1405	Eaton and Gasson, 2001
*cpd*	F:TGGTGGGTTATTTTTCAATTC R: TACGGCTCTGGCTTACTA	782	Eaton and Gasson, 2001
*cyl*	F: ACTCGGGGATTGATAGGC R: GCTGCTAAAGCTGCGCTT	688	Vankerckhoven et al., 2004
*efaAfm*	F:AACAGATCCGCATGAATA R: CATTTCATCATCTGATAGTA	735	Eaton and Gasson, 2001
*esp*	F:AGATTTCATCTTTGATTCTTGG R:AATTGATTCTTTAGCATCTGG	510	Vankerckhoven et al., 2004
*gelE*	F:ACCCCGTATCATTGGTTT R: ACGCATTGCTTTTCCATC	419	Sabia et al., 2008

### Statistical Analysis

The experiments were performed for each isolate in triplicate and the results were reported as mean ± SD of triplicates. The values of the mean and SD were calculated using Microsoft Excel.

## Results

### Molecular Identification of *E. faecium* Strains

Fifteen samples of rhizospheric soils were collected from different regions of India to isolate potential probiotic strains from LAB. Of these, five pure cultures named LR2, LR3, ER5, LR13, and VB1 were presumptively identified as LAB. The results of 16S rRNA gene sequencing and homology search using BLAST confirmed that these strains were *E. faecium*. The GenBank accession numbers of strains LR2, LR3, ER5, LR13, and VB1 are MK461568, MK461567, MK461570, MK461571, and MK461569, respectively.

### Phenotypic Testing of BSH and the *bsh* Gene

Appearance of a white zone of precipitation around the wells in bile-supplemented MRS indicated that *E. faecium* strain LR2 produced the enzyme BSH, while *E. faecium* strains LR3 ER5, LR13, and VB1 did not.

**FIGURE 1:**
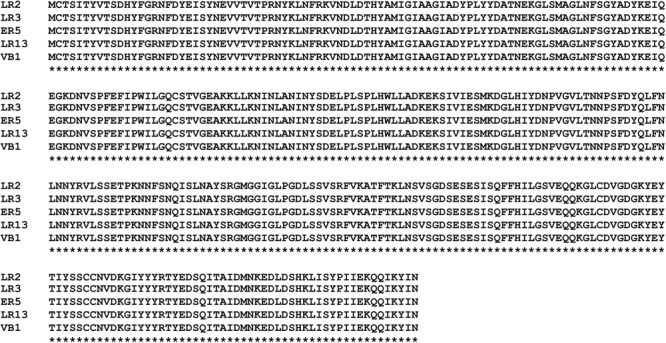
Multiple sequence alignment (MSA) of amino acid sequences of bile salt hydrolases (BSH) present in *E. faecium* LR2, LR3, ER5, LR13, and VB1 strains.

The primer pair (BF and BR) used for amplification of *bsh* resulted in the desired amplicon of 1.157 kbp in all the isolates. The purification, sequencing, and BLAST analysis of the PCR amplicons confirmed that these encoded for the *bsh* gene in *E. faecium* isolates. Multiple sequence alignment of the translated *bsh* revealed that the amino acid sequences of BSH of all *E. faecium* strains were identical (Figure 1).

### Cholesterol Assimilation

Though all the *E. faecium* isolates assimilated > 50% of the cholesterol present in the medium, the percentage of cholesterol assimilation increased in the presence of bile salt. Of all the strains, *E. faecium* LR13 showed a significantly higher capability than other strains to assimilate cholesterol from the medium, i.e., 76% in medium without bile salts and 98% in medium with bile salts (Table 2).

**Table 2:** Mean percentage cholesterol removal *in vitro* by *E. faecium* strains in MRS medium with and without bile salt.

*E. faecium* strain	Condition	Mean percentage cholesterol removal (%) ± SD
LR2	Cholesterol only	61.63 ± 0.33%
	Bile salt and cholesterol	91.70 ± 0.42%
LR3	Cholesterol only	60.57 ± 0.52%
	Bile salt and cholesterol	91.90 ± 0.15%
ER5	Cholesterol only	58.08 ± 2.10%
	Bile salt and cholesterol	85.70 ± 1.12%
LR13	Cholesterol only	75.97 ± 1.22%
	Bile salt and cholesterol	98.49 ± 0.60%
VB1	Cholesterol only	57.27 ± 0.64%
	Bile salt and Cholesterol	87.49 ± 0.49%

*For each sample, the mean of three values are presented ± SD.*

### Tolerance to Temperature, Sodium Chloride, and Lysozyme

All the five *E. faecium* isolates grew well in the temperature range of 28–45°C with optimum growth observed at 37°C. Similarly, all the isolates thrived in increasing concentrations of salt, with viabilities affected by not more than 0.5 log CFU as the salt concentration increased from 0.5 to 10%. Also, all the isolates showed high tolerance to lysozyme, with average percent survival being 75–80% even after 2 h of incubation (results not shown).

### Tolerance to Low pH and Simulated Gastric Juice

None of the *E. faecium* strain survived at pH 1.5 and 2.0. However, all the strains maintained their viability at pH 3.0 even after 3 h (Table 3). Similarly, all strains of *E. faecium* survived well in simulated gastric juice (pH 3.0) even after 3 h (Table 4).

**Table 3:** Viable cells (log_10_ cfu/ml) present in MRS broth at different pH and time intervals.

*E. faecium* strain	pH 1.5			pH 2.0			pH 3.0		
	0 h	1 h	3 h	0 h	1 h	3 h	0 h	1 h	3 h
LR2	4.36 ± 0.03	–	–	4.39 ± 0.03	–	–	4.48 ± 0.03	4.33 ± 0.03	4.14 ± 0.11
LR3	5.56 ± 0.08	–	–	5.63 ± 0.06	–	–	5.77 ± 0.02	5.57 ± 0.02	5.40 ± 0.02
ER5	4.56 ± 0.04	–	–	4.76 ± 0.04	–	–	4.96 ± 0.04	4.66 ± 0.04	4.56 ± 0.03
LR13	5.05 ± 0.02	–	–	5.25 ± 0.02	–	–	5.20 ± 0.02	5.16 ± 0.03	5.06 ± 0.02
VB1	5.16 ± 0.01	–	–	5.06 ± 0.01	–	–	5.46 ± 0.01	5.36 ± 0.04	5.16 ± 0.01

*For each sample, the mean of three values are presented ± SD.*

**Table 4:** Viable cells (log_10_ cfu/ml) present in simulated gastric juice (pH 3).

*E. faecium* strain	Viable cell count (log_10_cfu/ml)		
	0 h	2 h	3 h
LR2	8.48 ± 0.01	8.41 ± 0.02	8.36 ± 0.05
LR3	9.10 ± 0.02	9.08 ± 0.01	9.02 ± 0.07
ER5	8.90 ± 0.02	8.78 ± 0.01	8.62 ± 0.05
LR13	9.20 ± 0.02	9.18 ± 0.01	9.12 ± 0.03
VB1	8.68 ± 0.01	8.51 ± 0.02	8.36 ± 0.05

*For each sample, the mean of three values are presented ± SD.*

### Tolerance for Bile Salt and Antibiotic Susceptibilities

The number of colonies of all the isolates decreased as the concentration of bile salt increased from 0.06 to 1%, but all survived in concentration of 0.5% bile salt even after 2–4 h (Table 5).

**Table 5:** Survival of *E. faecium* strains after 4 h in the presence of 0.5% bile salts.

*E. faecium* strain	Log_10_ cfu/ml		
	0 h	2 h	4 h
LR2	8.51 ± 0.01	8.14 ± 0.01	8.14 ± 0.01
LR3	8.91 ± 0.02	8.84 ± 0.02	7.63 ± 0.01
ER5	8.46 ± 0.01	8.30 ± 0.01	8.23 ± 0.01
LR13	8.60 ± 0.03	8.50 ± 0.02	8.41 ± 0.02
VB1	8.40 ± 0.01	8.32 ± 0.02	8.32 ± 0.01

*For each sample, the mean of three values are presented ± SD.*

The antibiotic susceptibility profile of *E. faecium* isolates revealed that all the isolates were sensitive to gentamicin but resistant to kanamycin and erythromycin. Also, all the isolates were sensitive to amoxicillin, amoxicillin–clavulanic acid, and ciprofloxacin, except ER5 and VB1. Vancomycin resistance was observed in LR2, ER5, and VB1, while LR3 and LR13 were susceptible to this antibiotic (Table 6).

**Table 6:** Antimicrobial susceptibilities of *E. faecium* strains.

*E. faecium* strain	AMX (30)	AMC (30)	CIP (5)	(E) (15)	K (30)	GEN (10)	VA (30)
LR2	S	S	S	R	R	S	R
LR3	S	S	S	R	R	S	S
ER5	R	R	R	R	R	S	R
LR13	S	S	S	R	R	S	S
VB1	R	R	R	R	R	S	R

*Amoxycillin (AMX), amoxyclav (AMC), ciprofloxacin (CIP), erythromycin (E), kanamycin (K), gentamicin (GEN), vancomycin (VA); S, sensitive; I, intermediate; R, resistant. Numbers in parentheses indicate concentration of antibiotic in mcg/disk.*

### Biofilm-Forming Potential and Presence of Virulence Genes

All the *E. faecium* isolates showed a moderate ability to form biofilms at pH 4, 5, and 6 after time intervals of 24, 48, and 72 h (Table 7). It was also observed that none of the *E. faecium* isolates harbored any of the genes for virulence investigated in this study.

**Table 7:** Biofilm-forming potential of *E. faecium* strains.

*E. faecium* strain	OD_540 nm_								
	pH 4			pH 5			pH 6		
	24 h	48 h	72 h	24 h	48 h	72 h	24 h	48 h	72 h
LR2	0.20 ± 0.00 (M)	0.25 ± 0.01 (M)	0.26 ± 0.01 (M)	0.24 ± 0.01 (M)	0.25 ± 0.01 (M)	0.26 ± 0.01 (M)	0.24 ± 0.00 (M)	0.23 ± 0.01 (M)	0.25 ± 0.02 (M)
LR3	0.23 ± 0.01 (M)	0.27 ± 0.02 (M)	0.27 ± 0.01 (M)	0.25 ± 0.01 (M)	0.25 ± 0.01 (M)	0.25 ± 0.02 (M)	0.22 ± 0.02 (M)	0.23 ± 0.01 (M)	0.26 ± 0.03 (M)
ER5	0.23 ± 0.01 (M)	0.27 ± 0.01 (M)	0.27 ± 0.01 (M)	0.25 ± 0.01 (M)	0.25 ± 0.00 (M)	0.26 ± 0.01 (M)	0.25 ± 0.02 (M)	0.24 ± 0.04 (M)	0.25 ± 0.02 (M)
LR13	0.21 ± 0.02 (M)	0.26 ± 0.0 (M)	0.28 ± 0.0 (M)	0.25 ± 0.0 (M)	0.25 ± 0.03 (M)	0.25 ± 0.01 (M)	0.23 ± 0.01 (M)	0.22 ± 0.03 (M)	0.25 ± 0.01 (M)
VB1	0.24 ± 0.02 (M)	0.28 ± 0.0 (M)	0.29 ± 0.02 (M)	0.25 ± 0.01 (M)	0.26 ± 0.01 (M)	0.26 ± 0.01 (M)	0.25 ± 0.03 (M)	0.24 ± 0.03 (M)	0.29 ± 0.03 (M)
Control (medium alone)	0.10 ± 0.03	0.11 ± 0.01	0.13 ± 0.01	0.12 ± 0.01	0.12 ± 0.01	0.12 ± 0.01	0.10 ± 0.01	0.11 ± 0.00	0.12 ± 0.01

*For each sample, the mean of three values are presented ± SD. The letter inside the parentheses indicates moderate (M) biofilm producer.*

## Discussion

The present study is the first report on isolation of *E. faecium* from rhizospheric soils and assessment of their probiotic potential. The isolates were collected from sites located in northern, southern, and central regions of India. Rhizospheric soil represents an interesting ecological niche for isolation of probiotic bacteria as these bacteria directly co-evolve with plant pathogenic bacteria and fungi, and are competent to survive in the naturally microbe-enriched human GI tract. The five pure rhizospheric isolates were confirmed by 16S rRNA gene sequencing as *E. faecium* and evaluated for BSH activity, cholesterol-lowering capability, and probiotic properties.

It is important to evaluate the BSH activity of bacteria proposed as probiotics, because BSH has been recommended by the WHO as an important criterion for selection of probiotics. Results of the BSH phenotypic assay indicated that BSH was present only in strain LR2 and absent in other strains. However, PCR amplification and sequence analysis confirmed that *bsh* gene was present in all the strains. This non-concordance between the BSH phenotypic assay and presence of *bsh* gene indicates that phenotypic assay might not be a good indicator for screening of BSH activity in *E. faecium*. The presence of *bsh* in all rhizospheric *E. faecium* isolates was an interesting observation because BSH activity has rarely been reported from bacteria isolated from rhizospheric soil. Also, the fact that the amino acid sequences of BSH were identical in all the isolates indicates that these proteins are highly conserved in *E. faecium.*

All the rhizospheric *E. faecium* isolates showed a remarkable capability to assimilate cholesterol, which increased further in the presence of bile salts. In comparison to others, *E. faecium* isolate LR13 showed a significantly higher capability to assimilate cholesterol, i.e., 75% in medium without bile salts and 98% in medium with bile salts. This capability was much higher than the *in vitro* cholesterol removal capability of 42–55% reported previously for a probiotic *E. faecium* strain VC223 (Albano et al., 2018). This suggested that rhizospheric enterococci may be considered as good probiotic candidates, provided they satisfy other essential criteria for probiotic potential.

There are several characteristics that are essential for a probiotic strain to confer beneficial effects on the consumer, like adherence to the epithelial cells of the gut, tolerance to lysozyme, gastric juice, low pH, bile, and safety for human consumption. All the strains of *E. faecium* were salt tolerant and showed optimum growth at 37°C. Lysozyme disrupts the cell wall of Gram-positive bacteria. Since lysozyme is present in the human saliva; it is the first assault that probiotic bacteria must overcome to reach the human gut. It was observed that all the isolates of *E. faecium* grew well in high concentration of lysozyme (100 mg/l), even after contact with lysozyme for 2 h. Another attribute that probiotics need to possess to survive in the human gut is the low pH of the stomach, which might range from 1.5 to 2.0 and the gastric acid (pH 3.0) for at least 3–4 h. Though *E. faecium* isolates did not tolerate very low pH, i.e., 1.5 and 2.0, they survived at pH 3.0 for 3–4 h. Similarly, all the isolates survived in simulated gastric juice, even after 24 h. Thus, our results are in concordance with the results of previous studies (Banwo et al., 2013; Nami et al., 2014, 2018). After their passage through the stomach, probiotic bacteria have to combat the bile juices present in the intestine. A good tolerance to 0.3–0.5% bile helps the probiotic in easy colonization of the host gut (Luo et al., 2012). Thus, it was important to evaluate the survival of probiotic organisms in the presence of bile salts. All the isolates of *E. faecium* survived fairly in 0.5% bile salt, even after exposure for 2–4 h. Once inside the intestine, putative probiotics should adhere to intestinal mucosa, which facilitates transient colonization and impedes their removal by peristalsis. The biofilm potential of bacteria reflects their potential for cell adherence (Boris et al., 1997). All the isolates of *E. faecium* showed a moderate biofilm potential at pH 6, which is similar to the physiological pH of the intestine. The ability of probiotic bacteria to auto-aggregate helps in adherence to the epithelial cells and mucosal surfaces (Collado et al., 2008), while the ability to co-aggregate helps in excluding pathogenic bacteria from colonizing the human gut (Abushelaibi et al., 2017). Hence, these characteristics are also important classifiers for probiotics. All the isolates of *E. faecium* showed a moderate auto- and co-aggregation capabilities. None of the *E. faecium* isolates showed hemolytic or DNase activity, indicating their safety in potential probiotic preparations.

The safety of the enterococci isolates as good probiotics was also evaluated with regard to presence of virulence factors and antibiotic profile. The *E. faecium* isolates were evaluated for the presence of several virulence genes, *viz., esp, gelE, cyl, efaAfm, cpd, cob*, and *ccf.* As reported for another *E. faecium* probiotic strain SF68, none of the isolates harbored genes for virulence factors and, thus, may be considered as safe probiotic candidates (Kayser, 2003). The FAO/WHO (2002) recommended that, as a safety measure, the antibiotic resistance profile of a proposed probiotic should also be evaluated. The antibiotic susceptibility testing of *E. faecium* strains revealed that isolates ER5 and VBI were resistant to β-lactams, fluoroquinolones, kanamycin, and vancomycin, except gentamycin; hence, they might not be safe for human consumption. Many *Enterococcus* strains show an intrinsic resistance for β-lactam antibiotics (Lopes et al., 2005). Earlier studies reported a higher rate of aminoglycoside resistance in enterococcal strains isolated from dairy products (Hammad et al., 2015; Aspri et al., 2017); however, our results indicated that though all the rhizospheric soil isolates were resistant to kanamycin, they were susceptible to gentamycin. Thus, based on the susceptibility for the most common antibiotics used against *Enterococcus* infections (Favaro et al., 2014), it may be inferred that *E. faecium* isolates LR2 and LR13 might be considered as potential probiotic candidates.

## Conclusion

In this study, we have reported, for the first time, isolation and characterization of *E. faecium* from rhizospheric soil samples collected from different parts of India. Our results indicated that all the rhizospheric isolates showed a fair survival in the harsh conditions of the GI tract and did not harbor any of the virulence genes. Though all isolates showed potential for cholesterol removal, *E. faecium* isolate LR13 showed remarkable cholesterol removal capability and vancomycin susceptibility and possessed most of the desirable and essential attributes of a probiotic. Hence, it seems to be a fairly promising probiotic candidate that needs to be further evaluated in *in vivo* studies, especially for its hypo-cholesterolemic potential.

## Data Availability

The datasets generated for this study can be found in the GenBank, MK461568, MK461567, MK461570, MK461571, and MK461569.

## Author Contributions

NS designed the study. NS, AM, MK, and SM carried out the experiments. NS, MK, and JV drafted the manuscript.

## Conflict of Interest Statement

The authors declare that the research was conducted in the absence of any commercial or financial relationships that could be construed as a potential conflict of interest.

## Footnotes

1 www.ncbi.nlm.nih.gov/BLAST

2 www.expasy.org/translate

3 https://www.ebi.ac.uk/Tools/msa/clustalo/
